# High Cell Density Cultivation of *Paracoccus* sp. on Sugarcane Juice for Poly(3-hydroxybutyrate) Production

**DOI:** 10.3389/fbioe.2022.878688

**Published:** 2022-05-12

**Authors:** Ayyapruk Moungprayoon, Siriporn Lunprom, Alissara Reungsang, Apilak Salakkam

**Affiliations:** ^1^ Department of Biotechnology, Faculty of Technology, Khon Kaen University, Khon Kaen, Thailand; ^2^ Research Group for Development of Microbial Hydrogen Production Process from Biomass, Khon Kaen University, Khon Kaen, Thailand; ^3^ Academy of Science, Royal Society of Thailand, Bangkok, Thailand

**Keywords:** *paracoccus* sp., sugarcane, process optimization, high cell density cultivation, bioplastic

## Abstract

High cell density cultivation is a promising approach to reduce capital and operating costs of poly (3-hydroxybutyrate) (PHB) production. To achieve high cell concentration, it is necessary that the cultivation conditions are adjusted and controlled to support the best growth of the PHB producer. In the present study, carbon to nitrogen (C/N) ratio of a sugarcane juice (SJ)-based medium, initial sugar concentration, and dissolved oxygen (DO) set point, were optimized for batch cultivation of *Paracoccus* sp. KKU01. A maximum biomass concentration of 55.5 g/L was attained using the C/N ratio of 10, initial sugar concentration of 100 g/L, and 20% DO set point. Fed-batch cultivation conducted under these optimum conditions, with two feedings of SJ-based medium, gave the final cell concentration of 87.9 g/L, with a PHB content, concentration, and yield of 36.2%, 32.1 g/L, and 0.13 g/g-sugar, respectively. A medium-based economic analysis showed that the economic yield of PHB on nutrients was 0.14. These results reveal the possibility of using SJ for high cell density cultivation of *Paracoccus* sp. KKU01 for PHB production. However, further optimization of the process is necessary to make it more efficient and cost-effective.

## Introduction

Cane sugar is an important agro-industrial product of many countries, including Thailand. Due to its suitable climate and landscape (Jusakulvijit et al., 2021), Thailand is one of the largest sugarcane producers, ranking fourth after China, India, and Brazil, and is the second largest sugar exporter after Brazil (Food and Agricultureal Organization of the United Nations, 2022). During 2012 to 2017, Thailand produced, on average, 10.4 ± 0.7 million tonnes of raw sugar annually, and this increased to 15.1 ± 0.4 million tonnes in 2018 and 2019. However, the raw sugar export value had declined considerably from 0.5 million USD per tonne in 2012 to only 0.3 million USD per tonne in 2020 (Food and Agricultureal Organization of the United Nations, 2022). This decline, coupling with the reduced consumption of sugar due to health concerns (Van Rompay et al., 2015), has adversely impacted on the sugar industry. As a consequence, much efforts have been made to investigate the use of sugarcane or its related products to produce alternative valuable products to sustain the economics of the industry. In the field of biotechnology, as sugarcane juice (SJ) contains as high as 21 °Brix of total soluble solids (Sreedevi et al., 2018), with 13–15% sucrose (Singh et al., 2015), SJ is considered a highly feasible feedstock for microbial-derived products, including bioenergy, biochemicals, and bioplastics.

Poly (3-hydroxybutyrate) (PHB) is a biodegradable and biocompatible bioplastic belonging to the group of polyhydroxyalkanoates (PHAs). It is a natural polyester, with a repeating unit C_4_H_6_O_2_ (Yamane, 1993), that can be synthesized from various substrates and is accumulated as intracellular carbon and energy reserves in many microorganisms (Salakkam and Webb, 2018). This biopolymer has similar properties to polyethylene (PE) and polypropylene (PP), and therefore can substitute the petroleum-derived counterparts in a number of applications (Degli Esposti et al., 2021), ranging from mulch film for the agricultural sector and biodegradable packaging for the food sector to bio-scaffolds for medical applications (Kalia et al., 2021). Due to the increasing demand of bioplastics in recent decades, the market size of PHAs has been vigorously expanding. Recently, Vigneswari et al. (2021) reported that a compound annual growth rate of PHAs market per 10 years is 6.3%, and this would lead to an increase in global PHAs production from 70 million USD in 2015 to 119.15 million USD in 2025, with PHB as the main focus. This implies that PHB has a great potential to enhance the economic sustainability of the sugar industry.

To date, hundreds of microorganisms have been reported to be able to produce PHB. Among others, *Cupriavidus necator* is known to be one of the most promising ones, owing to its ability to consume a wide range of substrate, high growth rate, and high PHB production (Mozumder et al., 2014; Haas et al., 2017). However, this bacterium has a drawback of inability to utilize sucrose for growth and PHB production (Park et al., 2015; Arikawa et al., 2017). Therefore, it is necessary to explore sucrose-utilizing PHB producers for PHB production from SJ. In this regards, *Paracoccus* sp. emerges as a potential candidate. It is a facultative methylotrophic bacterium capable of utilizing a variety of substrates, including glycerol (Sawant et al., 2015), methanol, ethanol (Kojima et al., 2004), and sucrose (Nokhal and Schlegel, 1983), for PHB production. The bacterium uses invertase (EC 3.2.1.26) to hydrolyze sucrose into glucose and fructose and converts these sugars into pyruvate that is subsequently used in the PHB synthesis pathway similar to *C. necator* (Kojima et al., 2004). It can accumulate PHB to over 70% by weight when cultivated under stress conditions, e.g., nitrogen limitation (Sawant et al., 2015), and has previously been shown to produce comparable amount of PHB as compared with *C. necator* when grown on the same substrate, e.g., brown algae (*Laminaria japonica*) hydrolysate (Muhammad et al., 2020). PHAs production by *Paracoccus* sp. was previously investigated on several substrates, e.g., corn stover hydrolysate (Sawant et al., 2015), pure glycerol (Kumar et al., 2018), and defatted *Chlorella* sp. biomass (Khomlaem et al., 2020). However, the biomass productions in these studies were relatively low (9.1–24.2 g/L), resulting in low PHAs production. It was only recently that a high cell density cultivation (113 ± 0.92 g/L), with a satisfactory PHAs production, was reported, which was achieved through a continuous fermentation in a membrane bioreactor with glucose as the carbon source (Khomlaem et al., 2021). The results of these studies imply that PHB production by *Paracoccus* sp. are still in need of improvement. Besides, there has been no literature reports on PHB production from SJ by *Paracoccus* sp. Therefore, the present study was carried out aiming to use SJ as the sole carbon source for PHB production by *Paracoccus* sp. KKU01. The growth conditions, i.e., carbon to nitrogen (C/N) ratio of the SJ-based medium, initial total sugar concentration, and dissolved oxygen (DO) set point, were optimized to achieve the best cell growth. The optimum conditions were then used in fed-batch fermentation to produced PHB. Based on the results, a medium-based economic analysis was performed and the feasibility of PHB production by *Paracoccus* sp. was discussed.

## Materials and Methods

### Sugarcane Juice

Freshly harvested sugarcane stalk was purchased from local farmers in Khon Kaen, Thailand. It was milled to obtain the juice, which was subsequently concentrated by boiling to a total sugar concentration of *ca.* 600 g/L, and stored at −20°C until use. The concentrated sugarcane juice (SJ) contained sucrose as the major component. Potassium, magnesium, and calcium were the major elements in the concentrated SJ. Protein was also present in the juice, but at a very low level. The compositions of the concentrated SJ are given in Table 1.

**TABLE 1:** Compositions of concentrated sugarcane juice used in the present study.

Composition	Value
Sucrose (g/100 g-SJ)	49.5
Glucose (g/100 g-SJ)	5.61
Fructose (g/100 g-SJ)	5.2
Ash (g/100 g-SJ)	0.71
Protein (TN×6.25) (g/100 g-SJ)	0.47
Potassium (mg/100 g-SJ)	249.0
Magnesium (mg/100 g-SJ)	55.2
Calcium (mg/100 g-SJ)	47.6
Phosphorus (mg/100 g-SJ)	21.4
Iron (mg/100 g-SJ)	2.16
Sodium (mg/100 g-SJ)	0.73
Zinc (mg/100 g-SJ)	0.19
Copper (mg/100 g-SJ)	0.02

### Microorganism and Culture Conditions

*Paracoccus* sp. KKU01 was used as the PHB producer. It was isolated from a soil sample collected at a cassava starch production plant, Kaensiri Starch Co., Ltd., Phra Yuen, Khon Kaen, Thailand. The bacterium was screened and isolated using the method of Kitamura and Doi (1994), and was identified at the Thailand Bioresource Research Center (TBRC), Pathum Thani, Thailand, using 16S rDNA sequencing analysis. According to the TBRC report, two primers were used, i.e., 27F (5’-AGA GTT TGA TCM TGG CTC AG-3’) or 800R (5’-TAC CAG GGT ATC TAA TCC-3’) and 518F (5’-CCA GCA GCC GCG GTA ATA CG-3’) or 1492R (5’-TAC GGY TAC CTT GTT ACG ACT T-3’). The identification was carried out using the BLASTN program against 16S rDNA sequence database of validly published prokaryotes. The isolated strain was found to have 99.93% similarity to *Paracoccus denitrificans* (Accession no. MT491243). Preliminary study showed that *Paracoccus* sp. KKU01 could utilize glucose and sucrose for growth, and could accumulate PHB under nitrogen-limited conditions (unpublished data). The bacterium was maintained on nutrient agar, supplemented with 20 g/L of sucrose as the carbon source, at 30°C. Inocula were prepared by transferring one loop-full of the bacterium growing on nutrient agar into nutrient broth containing 20 g/L of sucrose, and incubating at 30 °C, 150 rpm, pH 6.9 for 15 h. PHB production medium was prepared following the formula of Kalaiyezhini and Ramachandran (2015). The medium comprised (per L) 5 g of K_2_HPO_4_, 0.5 g of KH_2_PO_4_, 1 g of MgSO_4_, and 2 ml of trace element solution. SJ (previously diluted to the designated total sugar concentration) and (NH_4_)_2_SO_4_ were used as the carbon and nitrogen sources, respectively. The trace element solution consisted of (per 1 L of 1 N HCl) 10 g of CaCl_2_·2H_2_O, 4.98 g of FeSO_4_·7H_2_O, 0.23 g of ZnCl_2_, 0.78 g of CuSO_4_·5H_2_O, 0.24 g of Na_2_MoO_4_·2H_2_O, and 0.61 g of MnSO_4_·H_2_O. The SJ, MgSO_4_, and trace element solution were sterilized separately at 121°C for 15 min, and aseptically mixed after being cooled to room temperature.

### Effect of Carbon to Nitrogen Ratio and Initial Sugar Concentration on Growth of *Paracoccus* sp. KKU01

Effects of carbon to nitrogen (C/N) ratio and initial sugar concentration were investigated in 500-mL Erlenmeyer flasks, with a working volume of 100 mL, using one-factor-at-a-time approach. In C/N ratio optimization, concentrated SJ was diluted with distilled water to a total sugar concentration of 30 g/L. Then, the diluted SJ was supplemented with minerals according to the mineral medium formula, and added with calculated amounts of (NH_4_)_2_SO_4_ to adjust the C/N ratios to 4 to 53 (by mass). The C/N ratio of four was selected based on the elemental compositions of *Paracoccus* sp. (CH_1.8_O_0.48_N_0.25_) reported by Van Verseveld and Stouthamer (1978), and the ratio of 53 was obtained from the ratio between carbon and nitrogen in the mineral medium used to grow *Paracoccus denitrificans* (Kalaiyezhini and Ramachandran, 2015). The medium was autoclaved at 121°C for 15 min, and mixed with sterile MgSO_4_ and trace element solutions before use. The fermentation was carried out at 30°C, pH 6.9, and 150 rpm, using an initial cell concentration of 0.2 unit of optical density at 600 nm (OD_600_). Effects of the initial sugar concentration in a range 30–150 g/L was subsequently examined using the optimum C/N ratio. The cultivation conditions were the same as described above. Samples were taken to follow the bacterial growth (in terms of OD_600_), and the consumption of total sugar during the fermentation. The experiments were conducted in triplicate, and the mean and standard deviation of the mean are reported.

### Effect of Dissolved Oxygen Level on Growth of *Paracoccus* sp. KKU01

Using the optimum C/N ratio and initial total sugar concentration, effects of dissolved oxygen (DO) level on growth of *Paracoccus* sp. KKU01 was examined. The fermentation was performed in batch mode in a 2-L bioreactor (Bioneer-neo-2L, B.E. Marubishi, Thailand), with a working volume of 1 L and an aeration rate of one vvm. The DO set point was varied in a range 10–50% of the oxygen saturation value. DO concentration was maintained at the designated level by automatic control of the agitation rate in the range 300 to 1,500 rpm. The bioreactor containing sterile medium was inoculated with *Paracoccus* sp. culture to an initial OD_600_ of 0.2 unit, and the fermentation was carried out at 30 ± 2°C, and pH 6.9 ± 0.2 (controlled using 10 N NaOH and 10 N HCl). Samples were taken regularly for the determination of cell dry mass (CDM) and total sugar concentrations.

### Fed-Batch Fermentation of *Paracoccus* sp. KKU01

Fed-batch fermentation was carried out in the 2-L bioreactor with an initial volume of 0.8 L. The fermentation temperature, pH, and DO set point were controlled at 30 ± 2°C, 6.9 ± 0.2, and 20%, respectively. The experiment was started as a batch fermentation with the optimum initial total sugar concentration (100 g/L), and was allowed to proceed until the sugar concentration decreased to below 30 g/L. Then, calculated amount of fresh medium (SJ that was previously supplemented with (NH_4_)_2_SO_4_ to attain a C/N ratio of 10) was fed into the bioreactor to increase the sugar concentration to around 100 g/L. The feeding of the medium was performed twice. The final volume of the medium was approximately 1 L. Samples were taken at 6-h intervals for the determination of CDM, total sugar, ammonia-nitrogen (NH_3_-N) and PHB concentrations. This experiment was conducted in duplicate.

### Medium-Based Economic Analysis

Economic yield (*EY*), defined as monetary unit (USD) of PHB produced per monetary unit (USD) spent in nutrients, was calculated using Eq. 1, where *Y* is the concentration of PHB (kg/L), *C* is the cost of each nutrient used in medium preparation (USD/kg), *N* is concentration of each nutrient (kg/L), and *I* is the selling price of PHB (USD/kg) (Bustos et al., 2004; Ma et al., 2021). It should be noted that the price of raw sugar (0.14 USD/kg) (Soccol et al., 2017) was used in the calculation due to the unavailability of concentrated SJ price in the literature. The selling price of PHB (3.50 USD/kg) was taken from Salakkam and Webb (2018), while the prices of other chemicals were taken from www.alfa.com (Supplementary Table S1).

$$EY=\frac{Y}{\sum \left(CN\right)}I$$ (1)

### Analytical Methods

Cell growth in terms of OD_600_ was measured using a UV-VIS spectrophotometer (UVmini-1240, Shimadzu, Japan). CDM (g/L) was measured gravimetrically after washing the cells twice with distilled water, and drying at 80°C until a constant weight was reached. Residual cell dry mass (RCDM) concentration was calculated as the difference between CDM and PHB concentrations. Total sugar concentration was determined using the phenol-sulfuric method (Dubois et al., 1956), using sucrose as the standard. Total sugar consumption rate at time interval was calculated by dividing total sugar consumed (g/L) by fermentation time (h). Sugar compositions of fermentation broth were determined using high performance liquid chromatography (HPLC) following the method of Khamtib and Reungsang (2012). Specific growth rate of the bacterium was estimated as the largest slope of a log-linear plot between OD_600_ and time. NH_3_-N concentration was determined using the phenate method (Baird et al., 2017), using ammonium sulfate as the standard. Polymer identification was conducted using Fourier transform infrared spectroscopy (FTIR) analysis at the Faculty of Associated Medical Sciences, Khon Kaen University, Thailand, using commercial poly (3-hydroxybutyrate) as the standard (Supplementary Figure S1). The PHB standard was purchased from Sigma-Aldrich (Cat. no. STBB9669V). Extraction of PHB from the biomass for the identification was performed using the method of Hahn et al. (1995). PHB concentration was determined by HPLC (Waters, United States), equipped with an Aminex HPX-87H column (Bio-Rad, United States) and a UV detector, using the conditions reported by Fernandes et al. (2011). The commercial PHB was used as the external standard. PHB production rate at time interval was calculated by dividing PHB concentration (g/L) by fermentation time (h). Kinetic parameters for biomass growth and PHB production were obtained by curve fitting using the modified Gompertz model (Eq. 2), where *Y*_m_ is the maximum CDM, RCDM, and PHB concentration (g/L), *R*_m_ is the maximum rate of CDM, RCDM, and PHB production (g/(L·h)), *e* is the Euler’s number (2.7183), *λ* is the lag time for the production of CDM, RCDM, and PHB (h), and *t* is the fermentation time (h). The Solver function of Microsoft Excel 2019 was used for curve fitting and estimation of the kinetic parameter values.

$$Y=Y_{\text{m}}\times \exp\left[-\exp\left[\frac{R_{m}\times e}{Y_{m}}\left(\lambda -t\right)+1\right]\right]$$ (2)

## Results and Discussions

### Effects of Carbon to Nitrogen Ratio and Initial Sugar Concentration on Growth of *Paracoccus* sp. KKU01

**FIGURE 1:**
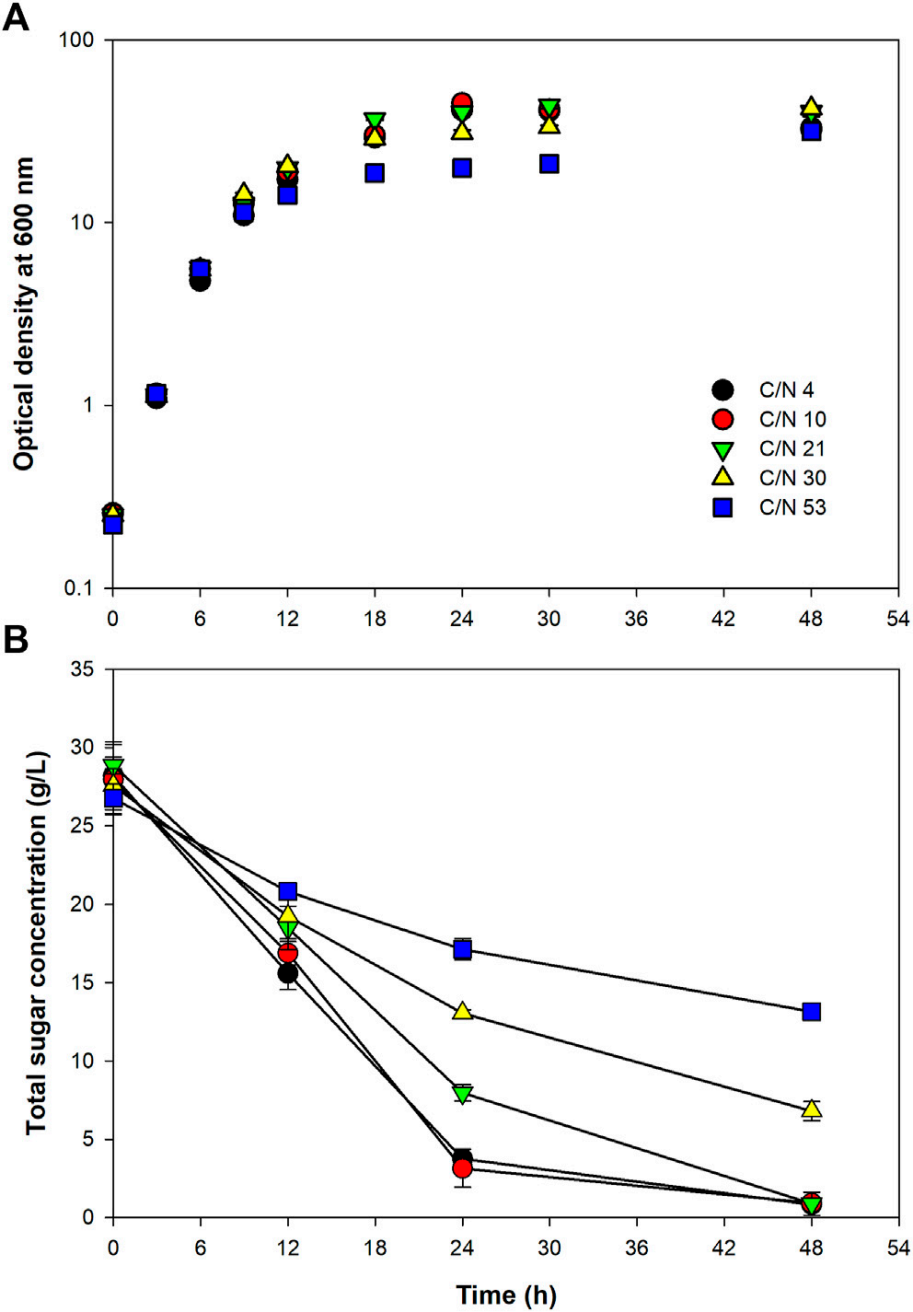
Growth of *Paracoccus* sp. KKU01 on sugarcane juice-based medium **(A)** and total sugar consumption **(B)** at C/N ratios of 4 to 53.

To achieve a high cell density cultivation, it is very important that the fermentation conditions are adjusted to support the best cell growth. It is well known that carbon and nitrogen are essential nutrients for microbial growth and cellular components synthesis, and the ratio of which directly affects the growth and functions of microorganisms. In the present study, C/N ratio of the fermentation medium was optimized to examine a proper balance between carbon and nitrogen sources. Growth curves of the bacterium at all the C/N ratios tested revealed that the bacterium grew rapidly without observable lag phase upon the inoculation. The exponential growth phase lasted for around 9–12 h (Figure 1A). Calculation of the specific growth rate (*µ*) during the first 9 h revealed that the bacterium grew equally well at all the C/N ratios tested; the value of *µ* varied in a narrow range of 0.424–0.484 1/h (Table 2). This implied that, at all the C/N ratios tested, nitrogen was sufficient for the bacterial multiplication during the early growth phase. However, after 9 h, the growth at C/N ratio of 53 started to be lower than that at other C/N ratios. At the C/N ratio of 53, with the initial total sugar concentration of 26.7 ± 1.0 g/L (equivalent to 11.23 g-carbon/L, assuming that all the sugar in SJ was sucrose), only *ca.* 1.0 g/L of (NH_4_)_2_SO_4_ was used in the medium (equivalent to 0.21 g-nitrogen/L). It could, therefore, be possible that most of the nitrogen was consumed during the exponential phase, resulting in insufficient nitrogen for the later stage of growth. As for the growths at other C/N ratios, both the *µ* and maximum OD_600_ values were very similar. It was initially presumed that C/N ratio of four would be suitable for cell growth since the C/N ratio of *Paracoccus* sp. has been reported to be around 4 (Van Verseveld and Stouthamer, 1978). Nevertheless, good growth could still be achieved at C/N ratios up to 30. Similar results have been reported by Sawant et al. (2015) that growths of *Paracoccus* sp. LL1 during the first 36 h were more or less the same at C/N ratios between 6.7 and 20, and further increasing the ratio to 40 led to a large drop of cell growth.

**TABLE 2:** Kinetic parameters of *Paracoccus* sp. KKU01 growth on sugarcane juice-based medium at C/N ratios of 4 to 53.

Parameter	C/N ratio				
	4	10	21	30	53
Maximum OD_600_	41.16 ± 0.83	45.12 ± 1.87	43.70 ± 1.70	42.17 ± 0.59	31.46 ± 0.88
Specific growth rate (1/h)	0.487 ± 0.002	0.513 ± 0.002	0.519 ± 0.002	0.515 ± 0.002	0.537 ± 0.004
Sugar consumption (%)∗	86.7 ± 1.1	88.8 ± 3.2	72.4 ± 2.0	75.4 ± 1.6	50.8 ± 3.6
Sugar consumption rate [g/(L·h)]∗	0.57 ± 0.05	0.56 ± 0.05	0.58 ± 0.01	0.43 ± 0.02	0.28 ± 0.03

∗calculated using the time at which cell growth reached the highest concentration

Determination of total sugar concentration revealed that sugar was rapidly consumed when the cells were grown at C/N ratios of 4 and 10, and this decreased with increasing C/N ratio up to 53 (Figure 1B and Table 2). It is well known that nitrogen is the macronutrient essential in the syntheses of various cellular components, *e.g.,* proteins, enzymes, DNA, and RNA (Zhou et al., 2007; Kampen, 2014; Kuypers et al., 2018). Therefore, the low availability of nitrogen at high C/N ratios could possibly lead to low microbial metabolic activity. Based on the results of the bacterial growth and sugar consumption (Figure 1), C/N ratio of 10 was considered optimum for growth of *Paracoccus* sp. KKU01, as at this C/N ratio, lesser amount of nitrogen source ((NH_4_)_2_SO_4_) was used compared with C/N ratio of 4. This was consistent with a report of Sawant et al. (2015) that C/N ratio of 13 was optimum for growth of *Paracoccus* sp. LL1.

**FIGURE 2:**
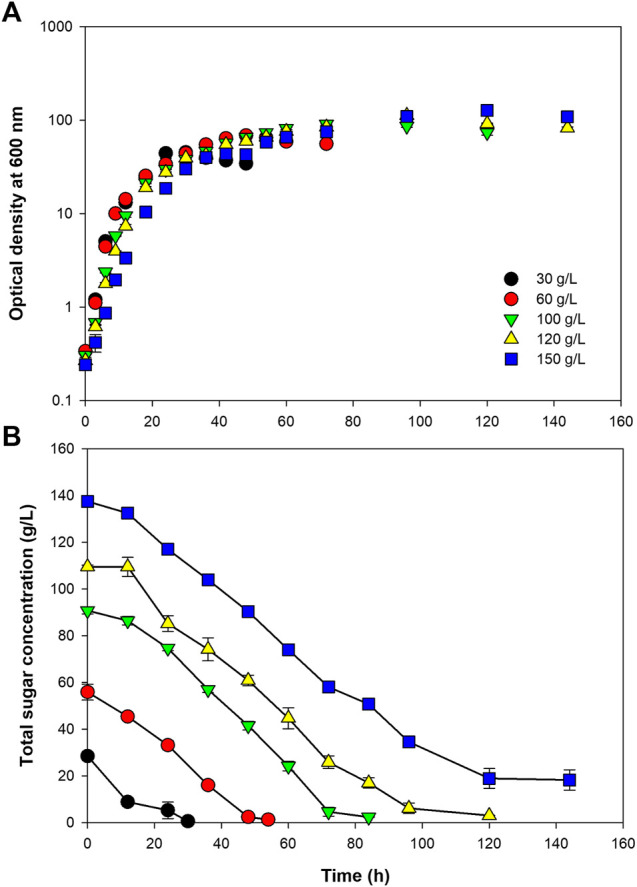
Growth of *Paracoccus* sp. KKU01 on sugarcane juice-based medium **(A)** and total sugar consumption **(B)** at initial total sugar concentration of 30–150 g/L.

Using the optimum C/N ratio of 10, *Paracoccus* sp. KKU01 was grown at initial sugar concentrations of 30–150 g/L. Figure 2A and Table 3 reveal that the final biomass concentration increased with increasing initial sugar concentration. However, the specific growth rate tended to decrease. The specific growth rate decreased by 74% when the initial sugar concentration was increased from 30 to 150 g/L. Likewise, biomass productivity and yield decreased by 36 and 39%, respectively. The lower growth observed at high initial sugar concentrations could be a result of increased osmotic pressures at higher solutes [sugars and (NH_4_)_2_SO_4_] concentrations, which can suppress cell growth (Sawant et al., 2015). The sugar consumption profile shown in Figure 2B reveal that the bacterium could completely consume sugar when grown at the initial sugar concentration as high as 120 g/L, while further increasing the initial sugar concentration to 150 g/L resulted in around 18.96 g/L of sugar remaining after the cultivation. Based on the results of biomass concentration, biomass yield, and sugar consumption rate, the initial sugar concentration of 100 g/L was selected as the suitable concentration for cell growth.

**TABLE 3:** Kinetic parameters of *Paracoccus* sp. KKU01 grown on sugarcane juice-based medium at initial total sugar concentrations of 30–150 g/L.

Parameters	Initial sugar (g/L)				
	30	60	100	120	150
Maximum biomass concentration (g/L)	14.73	28.57	44.03	49.38	54.23
Biomass productivity [g/(L·h)]∗	0.61 ± 0.11	0.60 ± 0.01	0.61 ± 0.01	0.51 ± 0.01	0.45 ± 0.01
Specific growth rate (1/h)	0.45 ± 0.003	0.43 ± 0.10	0.34 ± 0.015	0.32 ± 0.014	0.26 ± 0.038
Sugar consumption (%)∗	81.16 ± 12.94	95.56 ± 2.99	94.82 ± 2.08	94.32 ± 2.08	86.19 ± 3.13
Sugar consumption rate [g/(L·h)]∗	0.96 ± 0.15	1.11 ± 0.04	1.19 ± 0.02	1.08 ± 0.01	0.99 ± 0.04
Yield of biomass (g/g-sugar-consumed)∗	0.64 ± 0.02	0.54 ± 0.04	0.51 ± 0.01	0.48 ± 0.00	0.46 ± 0.01

∗calculated using the time at which cell growth reached the highest concentration

### Effect of Dissolved Oxygen on Growth of *Paracoccus* sp. KKU01

**FIGURE 3:**
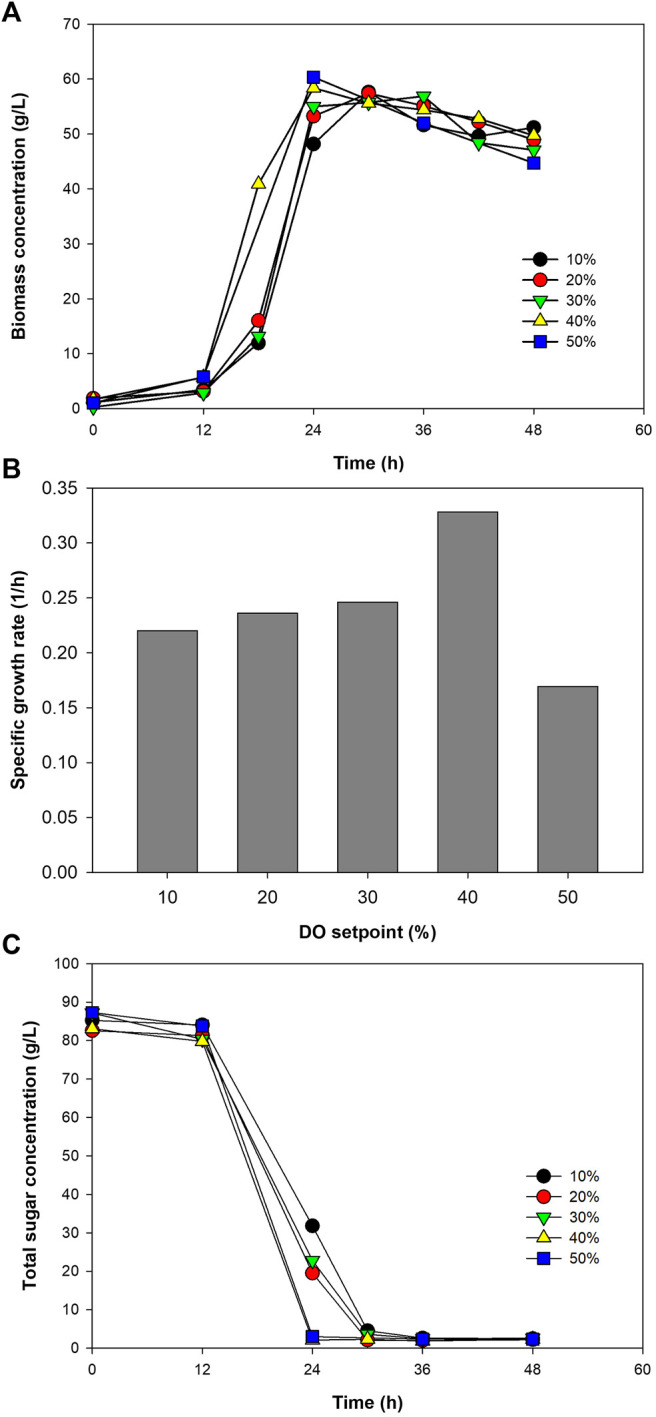
Growth profiles of *Paracoccus* sp. KKU01 on sugarcane juice-based media **(A)**, specific growth rate of the bacterium **(B)**, and total sugar consumption **(C)** at DO setpoints ranging from 10 to 50%.

Using the C/N ratio and initial sugar concentration of 10 and 100 g/L, respectively, growth of the bacterium was examined at DO set point ranging from 10 to 50% of the oxygen saturation level in the medium. The bacterial growth tended to increase as a function of DO set point (Figure 3A). However, *µ* only increased, from 0.220 to 0.328 1/h, when the DO set point was raised to 40%. At 50% DO set point, a large drop of *µ* to 0.169 1/h was observed (Figure 3B). This was considered due to the toxicity of oxygen at too high concentration. Although *Paracoccus* sp. is able to grow aerobically, the presence of oxygen at too high concentrations could lead to the formation of reactive oxygen species (ROS), *e.g.,* superoxide (O_2_^−^) and hydrogen peroxide (H_2_O_2_), inside the cells. These ROS can be accumulated to a concentration that overwhelms the antioxidant defenses of the cells, leading to cell inactivation. Some enzymes, particularly dehydratases and metalloenzymes, have also been reported to be inactivated by the presence of superoxide in the system (Baez and Shiloach, 2014).

Trend of sugar utilization correlated well with the bacterial growth. Increasing the DO set point resulted in higher rate of total sugar consumption (Figure 3C). At the DO setpoints of 10–30%, sugar was depleted at 30 h, while at 40% and 50% DO setpoints, more rapid sugar consumptions were observed, with the depletion of total sugar recorded at 24 h. Growth parameters of the bacterium at different DO setpoints are summarized in Table 4. As can be seen in the table, the bacterium consumed almost all the sugar (over 95%) in the system within 24–30 h, and the final biomass concentration and biomass yield were more or less the same at all the DO set points tested. It should be noted that the biomass productivity was higher at 40% and 50% DO setpoints because these were calculated using the time at which the total sugar was completely consumed. Considering that oxygen is inhibitory at 50% DO set point, and using 30% and 40% DO setpoint could lead to high operating costs, while using 10% DO set points led to low total sugar consumption rate, DO set point at 20% was selected for the subsequent experiments. The use of 20% DO setpoint was in accordance with previous studies using ≥20% DO setpoint to grow *Paracoccus* sp. for PHAs production (Kalaiyezhini and Ramachandran, 2015; Sawant et al., 2015; Kumar et al., 2018; Kumar and Kim, 2019).

**TABLE 4:** Kinetic parameters of *Paracoccus* sp. KKU01 cultivation on the optimum medium at DO setpoints ranging from 10% to 50%.

Parameters	DO setpoint				
	10%	20%	30%	40%	50%
Time at which total sugar was depleted (h)	30	30	30	24	24
Sugar consumption (%)∗	94.7	97.5	95.7	97.5	96.6
Sugar consumption rate [(g/(L·h)]∗	2.69	2.68	2.67	3.37	3.51
Maximum biomass (g/L)	56.5	55.5	55.5	56.7	59.3
Biomass productivity [g/(L·h)]∗	1.88	1.85	1.85	2.36	2.47
Yield of biomass (g/g-sugar-consumed)	0.70	0.69	0.69	0.70	0.70

∗calculated based on the time at which cell growth reached the highest concentration

### Fed-Batch Fermentation of *Paracoccus* sp. KKU01

**FIGURE 4:**
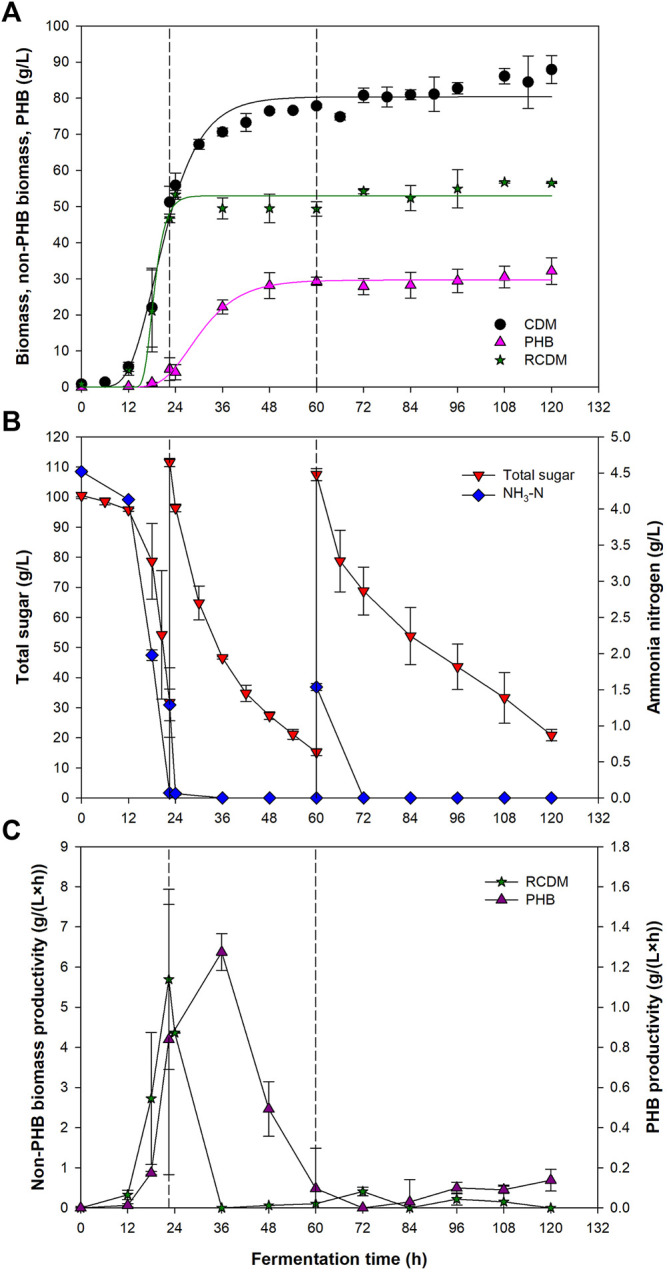
Cell dry mass (CDM), PHB, residual CDM **(A)**, total sugar and ammonia nitrogen concentrations **(B)**, and total sugar consumption rate and PHB production rate **(C)** during fed-batch fermentation of *Paracoccus* sp. KKU01 in SJ-based medium. The solid lines in **(A)** represent data predicted by the modified Gompertz model (Eq. 2). The dashed lines represent the time at which fresh medium was fed into the bioreactor.

Using the C/N ratio, initial sugar concentration, and DO set point of 10, 100 g/L, and 20%, respectively, *Paracoccus* sp. KKU01 was cultivated in batch mode until total sugar concentration decreased to below 30 g/L, then fresh medium was fed into the bioreactor to start the fed-batch cultivation. Figure 4A shows that the biomass concentration increased slowly during the first 12 h. After that, the cells grew rapidly, reaching 51.2 g/L at 22.5 h, corresponding to the large drops of total sugar and NH_3_-N concentrations to 31.7 and 0.07 g/L, respectively (Figure 4B). PHB was detected at as early as 12 h at 0.2 g/L, indicating the commencement of the PHB accumulation phase. Accordingly, fresh medium was fed into the bioreactor at 22.5 h to provide carbon source for PHB synthesis. It is noteworthy that although nitrogen deficiency is preferable for PHB production, complete deficiency of nitrogen might not always be supportive for PHB production as microbial PHB synthetic activities could be damaged (Suzuki et al., 1986). Therefore, nitrogen should be provided in the feed to support the catalytic activity and maintenance of the cells (Khanna and Srivastava, 2006; Norhafini et al., 2019). For this reason, the present study used fresh medium (SJ supplemented with (NH_4_)_2_SO_4_) as the feeding solution. Provision of nitrogen source in the feed has been shown to be beneficial for PHB production in several PHB producers, e.g., *Pseudomonas extorquens* sp. K. (Suzuki et al., 1986), *Ralstonia eutropha* NRRLB14690 (Khanna and Srivastava, 2006), *Wautersia eutropha* NRRLB14690 (Khanna and Srivastava, 2008), *Cupriavidus malaysiensis* USMAA1020 (Norhafini et al., 2019), *Halomonas venusta* (Stanley et al., 2018), and *Paracoccus* sp. LL1 (Khomlaem et al., 2021). Noted that *R. eutropha* and *W. eutropha* are now known as *Cupriavidus necator*. Following the feeding, total sugar and NH_3-_N concentrations were 111.5 g/L, and 1.3 g/L, respectively. NH_3_-N was depleted shortly after the feeding, implying that nitrogen was required by the cells even if these were in the PHB accumulation phase. With the depletion of NH_3_-N, excess carbon and limited nitrogen conditions were achieved, which are favorable for PHB synthesis by *Paracoccus* sp. (Kojima et al., 2004). Vigorous PHB accumulation proceeded until 48 h, attaining 28.1 g/L of PHB. At which time, total sugar and NH_3_-N concentration reduced to 27.3 and 0 g/L, respectively. The second feeding was performed at 60 h when total reducing sugar concentration decreased to 15.2 g/L, which increased the total sugar and NH_3_-N concentration to 107.5 and 1.5 g/L, respectively. However, neither significant biomass growth nor PHB production were observed after the second feeding. At the end of the fermentation, the CDM, RCDM, and PHB concentration were 87.9, 56.5, and 32.1 g/L, respectively. The PHB content in the cell was 36.2% and PHB productivity was 0.27 g/(L·h).

**FIGURE 5:**
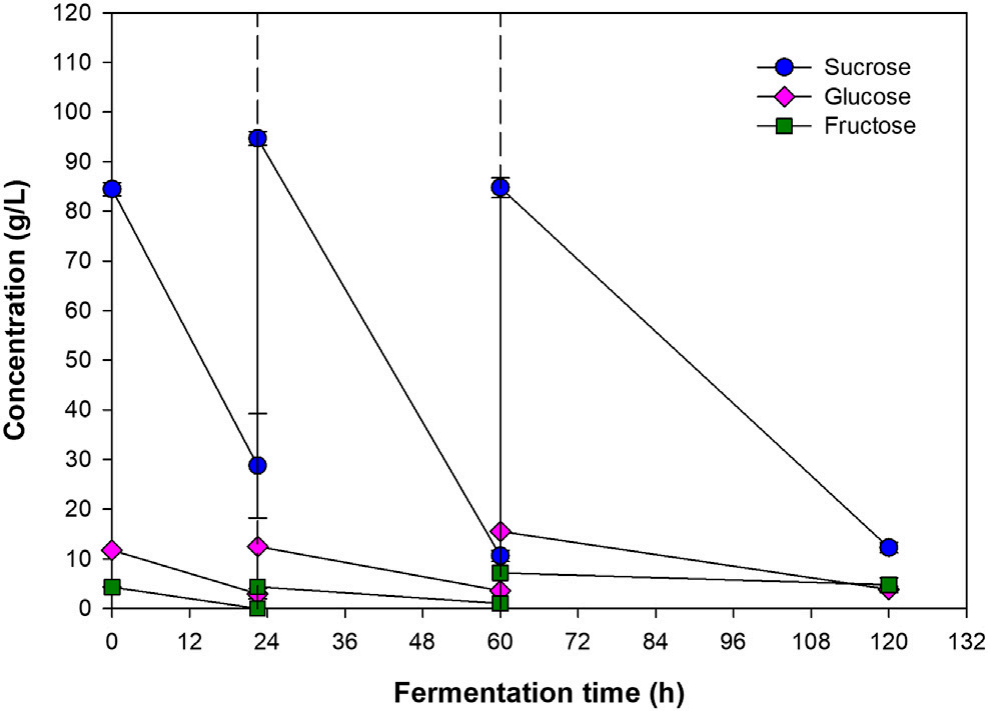
Concentrations of sucrose, glucose, and fructose at the beginning, before and after first and second feedings of the feed, and the end of the process. The dashed lines represent the time at which fresh medium was fed into the bioreactor.

Profiles of CDM, RCDM, and PHB concentrations were fitted well with the modified Gompertz model (Eq. 2), which is commonly used to predict population growth and bioproduct formation (Zeb et al., 2019). The kinetic parameters for CDM, RCDM, and PHB, are given in Table 5. Based on the curve fittings (Figure 4A), it can be seen that RCDM (non-PHB biomass) concentration increased rapidly during 12–22.5 h, coinciding with the rapid sugar consumption before the first feeding of fresh medium, then the concentration stayed relatively constant until the end of the fermentation. The total sugar consumption rate shown in Figure 4C confirmed that the sugar was mainly consumed for biomass growth during this period. On the other hand, vigorous PHB production occurred after 18 h and lasted until 48 h. The PHB production rate decreased afterward and no further production was observed until the end of the fermentation. The profile of PHB production rate (Figure 4C) confirmed that most of the sugar present the bioreactor after the first feeding was used for PHB synthesis. Analysis of the sugar compositions in the fermentation broth at different times during the process, i.e., 0 h, 22.5 h (before and after the first feeding), 60 h (before and after the second feeding), and 120 h, revealed that the bacterium utilized sucrose, glucose, and fructose for growth and PHB production (Figure 5). However, it was interesting that there were only little biomass growth and PHB production after the second feeding at 60 h, yet the sugar was continually consumed. This was possibly due to the utilization of the sugar in other metabolic pathways for cell maintenance and for syntheses of other products. Astaxanthin, for instance, could be produced simultaneously with PHAs by *Paracoccus* sp. According to a report of Khomlaem et al., 2021, up to 8.51 ± 0.20 and 10.2 ± 0.24 mg/L of intracellular and extracellular astaxanthin were observed during a continuous fermentation of *Paracoccus* sp. LL1 in a membrane bioreactor. Additionally, secondary metabolites such as polyketides, bacteriocins, and non-ribosomal peptides might also be produced (Leinberger et al., 2021). Since the production of PHB was not observed after 60 h (Figure 4A), the process could be terminated at this time, which would improve the PHB productivity to 0.49 g/(L·h).

**TABLE 5:** Kinetic parameters for CDM, RCDM, and PHB productions predicted by modified Gompertz model (Eq. 1).

Parameters	CDM	RCDM	PHB
Maximum value (*Y*_m_, g/L)	80.4	53.0	29.7
Maximum rate of production [*R*_m_, g/(L·h)]	4.6	10.0	1.5
Lag time (*λ*, h)	12.1	15.9	20.6
Coefficient of determination (*R* ^2^)	0.9837	0.9783	0.9927

With the total sugar consumption of 251.6 g/L and the production of 32.1 g-PHB/L, a PHB yield was calculated to be 0.13 g-PHB/g-sugar. This was around 26% of the biochemical stoichiometric yield 0.5 g-PHB/g-sucrose reported by Yamane (1993). Based on the results of total sugar consumption, it was also possible to estimate the quantity of carbon utilized for biomass growth and PHB production, using an approach similar to that proposed by Bormann and Roth (1999). Since the elemental formula for *Paracoccus* sp. is CH_1.8_O_0.48_N_0.25_ (molecular weight 25.03 g/mol) (Van Verseveld and Stouthamer, 1978; Araujo-Granda et al., 2020), and the monomer of PHB is C_4_H_6_O_2_ (molecular weight 86 g/mol) (Yamane, 1993), carbon accounts for 47.9% and 55.8% of the biomass and PHB, respectively. With 56.5 g/L of RCDM and 32.1 g/L of PHB, the carbon in RCDM and PHB were calculated to be 27.1 and 17.9 g/L, respectively. This demonstrated that around 45 g/L of carbon, equivalent to 42.5% of the total carbon consumed, could be referred to RCDM and PHB. The elemental formula for *Paracoccus* sp. KKU01 accumulating 36.6% PHB could also be calculated as seen in Eq. 3.

$$\left(\frac{56.5}{25.03}\right){\text{CH}}_{1\text{.}8}{\text{O}}_{\text{0}\text{.}48}{\text{N}}_{\text{0}\text{.}25}+\left(\frac{32.1}{86}\right){\text{C}}_{4}{\text{H}}_{6}{\text{O}}_{2}\to {\text{C}}_{3\text{.}75}{\text{H}}_{6\text{.}3}{\text{O}}_{1\text{.}84}{\text{N}}_{\text{0}\text{.}56}$$ (3)

### Feasibility of PHB Production by *Paracoccus* sp. KKU01

The key results for PHB production from the present study along with those from recent publications are shown in Table 6. It can be seen that PHAs production varies depending on the microbial strain, substrate, and fermentation mode and conditions. It is obvious that *Cupriavidus necator* could be grown to high cell concentrations (148–164 g/L) and produced PHB at high concentrations (109–125 g/L) and productivities [1.5–3.1 g/(L·h)]. Nevertheless, *Paracoccus* sp. LL1 could also be grown to a high cell density (113 g/L) using a continuous fermentation process with a cell retention system (Khomlaem et al., 2021), though PHB production and productivity were lower. In the present study, *Paracoccus* sp. KKU01 was grown to a high cell density of 87.9 g/L and produced 32.1 g/L of PHB. These figures, although not the highest, were in the upper range among the results compared, suggesting that *Paracoccus* sp. KKU01 is a potential PHB producer from SJ. Better still, the bacterium may be used with other low-cost feedstocks containing sucrose, *e.g.,* cane molasses (Palmonari et al., 2020) and fruit wastes (Choi et al., 2015; Nanda et al., 2016), which would be beneficial in reducing raw material cost. However, despite the promising results, the medium-based *EY* calculation revealed that the process used in the present study gave a low *EY* of 0.14, indicating that the revenue of PHB production is less than the cost of the medium. It is therefore necessary to further optimize the process to increase PHB production and to reduce the cost of medium. Increasing PHB production may be achieved by precise control of oxygen supply during the process (Blunt et al., 2018), by the use of genetic engineering to overexpress the *pha* genes involved in PHB synthesis (Khetkorn et al., 2016), or by using an effective process configuration such as multi-stage chemostat continuous fermentation (McAdam et al., 2020). The use of nutrients limitation strategy could also be further investigated with other nutrients. The limitation of phosphorus in the medium has been demonstrated to be effective in enhancing PHAs production in *C. necator* DSM 545 (Grousseau et al., 2014), while this improved the growth of *Burkholderia sacchari* LMG19450 on xylose (Oliveira-Filho et al., 2020). Double nutrients limitation, e.g., nitrogen-oxygen limitation, might also be applicable as this has been shown to enhance PHAs production in *Halomonas boliviensis* (Garcia-Torreiro et al., 2016). However, the expenditure of the process must be taken into consideration. Optimization of the feed composition, as well as feeding strategy, could also be performed to further enhance the PHB production. On the other hand, since the *EY* calculation showed that the cost of K_2_HPO_4_ accounted for over 80% of the total medium cost, medium optimization is worth the investigation. SJ already contains appreciable concentrations of potassium and phosphorus as shown in Table 1 and reported by De Souza et al. (2015). Therefore, it might be possible to reduce the concentration of K_2_HPO_4_ in the medium to reduce cost of medium preparation.

**TABLE 6:** Polyhydroxyalkanoates (PHAs) production by bacteria.

Microorganism	Substrate	Fermentation mode	Types of PHA	Biomass (g/L)	PHA (g/L)	PHA content (%)	PHAs yield (g/g)	PHAs productivity (g/L·h)	References
*Burkhoderia sacchari*	Waste paper hydrolysate	Batch	PHB	3.63	1.6	44.2	0.15	0.03	Al-Battashi et al. (2019)
*B. sacchari* IPT101	Ensiled grass press juice	Fed-batch	mcl-PHA	44.5	13.8	35	−	0.38	Cerrone et al. (2015)
*Cupriavidus necator* DZM 545	Glucose	Fed-batch	PHB	164	125	76.2	−	2.03	Mozumder et al. (2014)
*C. necator* H16	Glucose	Fed-batch in membrane bioreactor	PHB	148	113	76	0.33	3.1	Haas et al. (2017)
*C. necator* H16	Palm olein	Fed-batch	PHB	161	109	68	−	1.51	Zainab and Sudesh (2019)
*C. necator* DSM 4058	Crude glycerol	Fed-batch	PHB	28.86	24.75	85.76	0.32	0.2	Salakkam and Webb (2018)
*C. necator* NCIMB 11599	Brown algae hydrolysate	Fed-batch	PHB	8.3	4.1	49.4	−	0.04	Muhammad et al. (2020)
*Ralstonia eutropha H16*	Acetic, propionic, butyric acids	Fed-batch	PHBV	112	93.5	83.1	−	2.13	Huschner et al. (2015)
*Escherichia coli* (genetically modified)	Sucrose	Batch	PHB	9.84	3.76	38.21	−	0.13	Sohn et al. (2020)
*Pseudomonas chlororaphis* IMD555	Ensiled grass press juice	Fed-batch	mcl-PHA	37.5	3.75	10	−	0.1	Cerrone et al. (2015)
*P. chlororaphis* 555	Waste cooking oil	Fed-batch	mcl-PHA	73	13.9	19.04	−	0.29	Ruiz et al. (2019)
*P. putida* LS46	Octanoic acid	Fed-batch	mcl-PHA	28.9	17.7	60.6	0.33	0.66	Blunt et al. (2019)
*Paracoccus* sp. LL1	Glucose	Continuous with a cell retention system	PHBV	113	43.9	38.8	−	0.91	Khomlaem et al. (2021)
*Paracoccus* sp. LL1	Corn stover hydrolysate	Batch	PHB	14.8	9.71	72.4	0.25	0.13	Sawant et al. (2015)
*Paracoccus* sp. LL1	Brown algae hydrolysate	Fed-batch	PHBV	13.46	4.98	37	−	0.04	Muhammad et al. (2020)
*Paracoccus* sp. LL1	Defatted Chlorella biomass hydrolysate	Batch	PHBV	9.1	3.62	39.8		0.13	Khomlaem et al. (2020)
*Paracoccus* sp. KKU01	Sugarcane juice	Fed-batch	PHB	87.9	32.1	36.6	0.13	0.27	This study

mcl, medium chain length; PHBV, polyhydrolxybutyrate-*co*-valerate.

## Conclusion

SJ was used as the sole carbon source to grow *Paracoccus* sp. KKU01 to a high cell density for PHB production. The growth conditions, i.e., C/N ratio of SJ-based medium, initial sugar concentration, and DO set point were optimized, yielding the maximum biomass concentration of 55.5 g/L at C/N ratio of 10, initial sugar concentration of 100 g/L, and 20% DO set point. Fed-batch cultivation carried out using the optimum conditions gave a final biomass concentration of 87.9 g/L, with PHB content, concentration, and yield of 36.2%, 32.1 g/L, and 0.13 g/g, respectively. Overall, the present study demonstrates the feasibility of using SJ for high cell density cultivation of *Paracoccus* sp. KKU01 for PHB production. However, the medium-based economic analysis indicates the needs for further optimization of the process, so as to make it more efficient and cost-effective.

## Data Availability

The original contributions presented in the study are included in the article/Supplementary Material, further inquiries can be directed to the corresponding author.
