# Supplementary material for: High Cell Density Cultivation of Paracoccus sp. on Sugarcane Juice for Poly(3-hydroxybutyrate) Production
Source: Front Bioeng Biotechnol. 2022 May 12;10:878688. doi: 10.3389/fbioe.2022.878688 (PMC9133739; doi:10.3389/fbioe.2022.878688)
Supplement: Supplementary file 1 [file DataSheet1.docx]

Supplementary Materials

**Table S1** Costs of nutrients in the culture media formulation

| Nutrients | Product number | Price (USD/kg) |
| --- | --- | --- |
| (NH_4_)_2_SO_4_* | - | 0.24 |
| K_2_HPO_4_ | 11593 | 91.2 |
| KH_2_PO_4_ | A12142 | 71.6 |
| CaCl_2_·2H_2_O | 12312 | 65.5 |
| FeSO_4_·7H_2_O | A15178 | 31.9 |
| ZnCl_2_ | A16281 | 28.3 |
| CuSO_4_·5H_2_O | A11262 | 25.3 |
| Na_2_MoO_4_·2H_2_O | 12214 | 396.0 |
| MnSO_4_·H_2_O | A11809 | 68.8 |
| HCl | 33257 | 25.5 |
| (NH_4_)_6_Mo_7_O_7_ | 11831 | 265.5 |
| Na_2_B_4_O_7_ | 12305 | 18.8 |
| MgSO_4_·7H_2_O | A14491 | 17.5 |

Cost of nutrients as of 20^th^ October 2021 from Alfa Aesar, Thermo Fisher Scientific, United Kingdom (www.alfa.com)

*Cost of (NH_4_)_2_SO_4_ (Fertilizer grade) as of 16^th^ February 2022 from the Office of Agricultural Economics (www.oae.go.th)


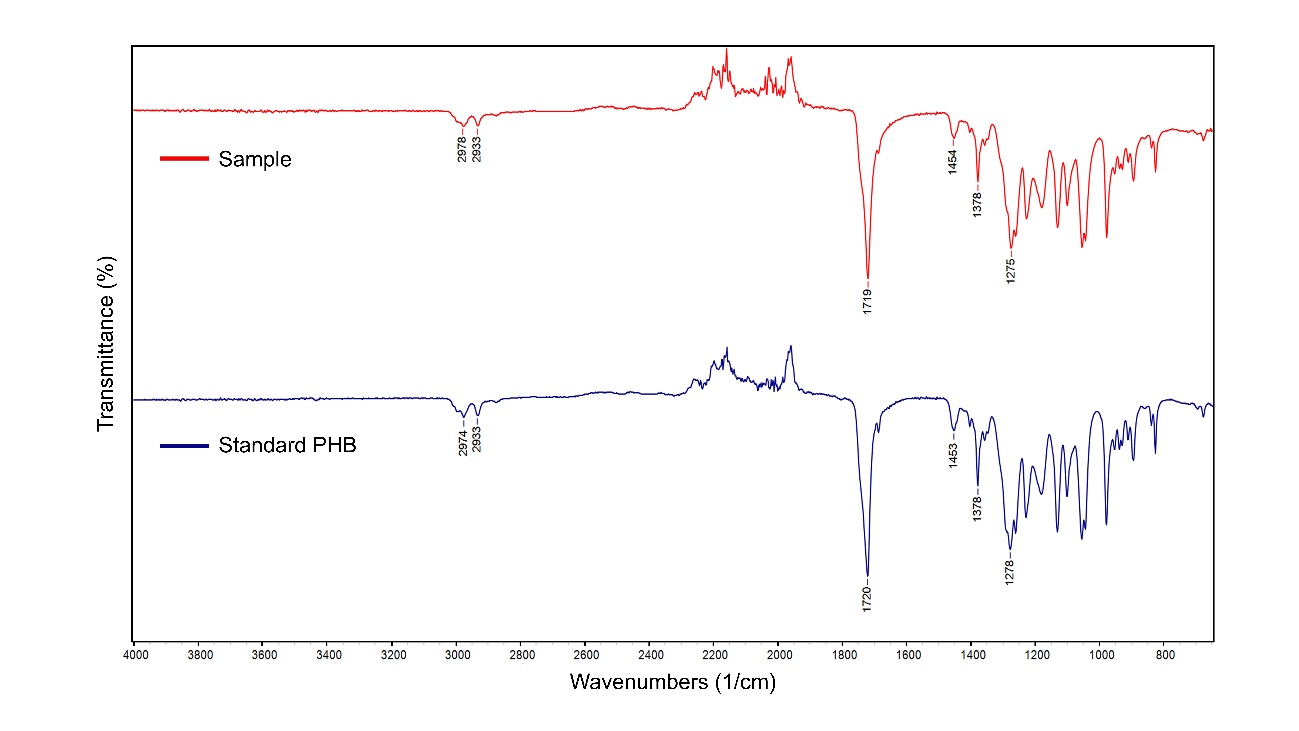


Fig. S1 Fourier transform infrared (FTIR) spectrums of standard PHB and PHB extracted from *Paracoccus* sp. KKU01.
